# Physical and Psychological Effects of Bariatric Surgery on Obese Adolescents: A Review

**DOI:** 10.3389/fped.2020.591598

**Published:** 2021-01-27

**Authors:** Cherie A. Roberts

**Affiliations:** ^1^Department of Nutrition, Columbia University, New York, NY, United States; ^2^Department of Biomedical Sciences, Touro College of Osteopathic Medicine-Harlem, New York, NY, United States; ^3^Department of Osteopathic Medicine, Touro College of Osteopathic Medicine-Harlem, New York, NY, United States

**Keywords:** bariatric (weight-loss) surgery, obesity, adolescent obesity, gastric bypass, sleeve gastrectomy, weight loss (%), gastric bypass (RYGB), obstructive sleep apnea

## Abstract

The worldwide obesity crisis is not isolated to adults; rather, obesity in adolescents has reached epidemic levels as well. Bariatric surgery continues to be one of the most effective treatments for obesity, both in adults and adolescents, with new evidence continually emerging; however, research surrounding outcomes of these procedures in younger patients is limited in comparison with data available for adults. Further, it is important to examine psychological aspects of obesity in adolescents, as well as effects of surgery on mental health endpoints. Conditions such as anxiety, depression, anger, and disruptive behavior show increased prevalence among obese adolescents, but minimal research exists to examine changes in such conditions following bariatric surgery. Additionally, there is growing evidence of a bidirectional relationship between sleep (quality; disorders) and the development of obesity, and the effects of this relationship are particularly pronounced in the vulnerable adolescent population. This review aims to compile and discuss the results of literature within the last 5 years with regard to overall efficacy of bariatric surgery specifically in adolescent patients in terms of weight and body mass index (BMI) reduction, hormonal changes, and co-morbidity resolution, as well as data surrounding sleep and psychological outcomes. Race, ethnicity, and socioeconomic status were also examined. From this review, we conclude that current research supports bariatric surgery in adolescents as an effective method of treatment for obesity and related co-morbidities; however, minimal long-term data exists to adequately assess efficacy and trends into adulthood. These areas are ripe for future study.

## Introduction

Clinical obesity in adults is characterized by a body mass index (BMI) more than 30 kg/m^2^, and it is the result of multiple, combined factors such as genetics, cultural influences, socioeconomic status (SES), and mental health (1). Over the last four decades, worldwide obesity rates have nearly tripled, and in 2016, close to two billion adults over the age of 18 were overweight or obese—roughly 40 percent of the global population (2). Further, obesity increasingly impacts younger individuals, with 340 million overweight or obese children and adolescents under the age of 19 (2). In children and adolescents, ages 5 to 19, a BMI-for-age more than one standard deviation higher than the World Health Organization (WHO) growth median is considered overweight, and greater than two standard deviations is considered obese (2). Complicating matters further is the myriad of co-morbidities associated with obesity (1). Issues such as glucose intolerance, dyslipidemia, hypertension, type 2 diabetes, kidney failure, chronic liver disease, and osteoarthritis are just some of the conditions associated, and research links each with obesity in a potentially bidirectional, causal relationship (1, 3).

Bariatric surgery is not only the most successful clinical intervention for obesity, but also the most cost-effective when considered long-term (4, 5); however, until recently, data regarding its effectiveness in obese adolescents has been scarce. When obesity begins in childhood or adolescence, the onset of co-morbidities often does as well, leading to the increased severity of adverse health outcomes as patients age. Research suggests that obesity in young adulthood could lead to as much as a 64-percent increase in adult mortality, independent of sex, race, or adult obesity status (6), including a nearly two-fold rise in coronary heart disease mortality (7). Though many behavioral and lifestyle interventions have been developed and evaluated, recent data shows that environmental and policy programs, such as advertisement regulation, sugar-sweetened beverage taxation, and restaurant portion reduction; as well as lifestyle changes, including physical activity and increased socialization, are most effective with children under the age of nine (8–10). This leaves the adolescent population at continued risk for worsening obesity without more intensive clinical interventions.

Additionally, beyond the physical consequences and co-morbidities in these young patients, obesity also has ramifications with regard to mental health, sleep patterns and disorders, and educational and professional successes, both during adolescence and into adulthood (11–20). In an age where teenagers live to a large extent in a virtual social environment, obese adolescents often experience a perpetual stream of cyber-bullying, fat-shaming, and messages about the “ideal” body type. Such stimuli lead to increased rates of depression and anxiety, body dysmorphia disorder, stress, and sleep disruption, and adolescent girls are often more heavily affected, leading to alterations in mental health that last long into their adult years (12–17). With such a diverse pool of influencing factors, it is difficult to determine whether obesity affects the mental health of these teenagers, or if mental health issues lead to increased obesity. Regardless of directionality, obese adolescents suffer disproportionately from a myriad of mental health issues, such as depression, anxiety, bullying, and eating disorders, as well as decreased self-esteem and lower health-related quality of life (16). Addressing these issues earlier in the life cycle is key to preventing future negative impacts, and the available evidence suggests mental health improvement from surgical weight loss procedures.

Bariatric surgery continues to garner support as one of the most effective treatment methods for obesity, with long-term, sustainable results, and a measurable impact on lifetime health expenditures and related co-morbidities (21–39). Klebanoff et al. (4) noted a significant increase of 0.199 in Quality Adjusted Life Years (QALYs) at 3 years post-bariatric surgery, and lifetime cost-effectiveness beginning at 5 years post-intervention. Any surgical procedure innately entails risk; however, evidence of long-term physical and psychological benefits, as discussed herein, supports the notion that the rewards, in this case, outweigh the risks.

## Materials and Methods

**Figure 1:**
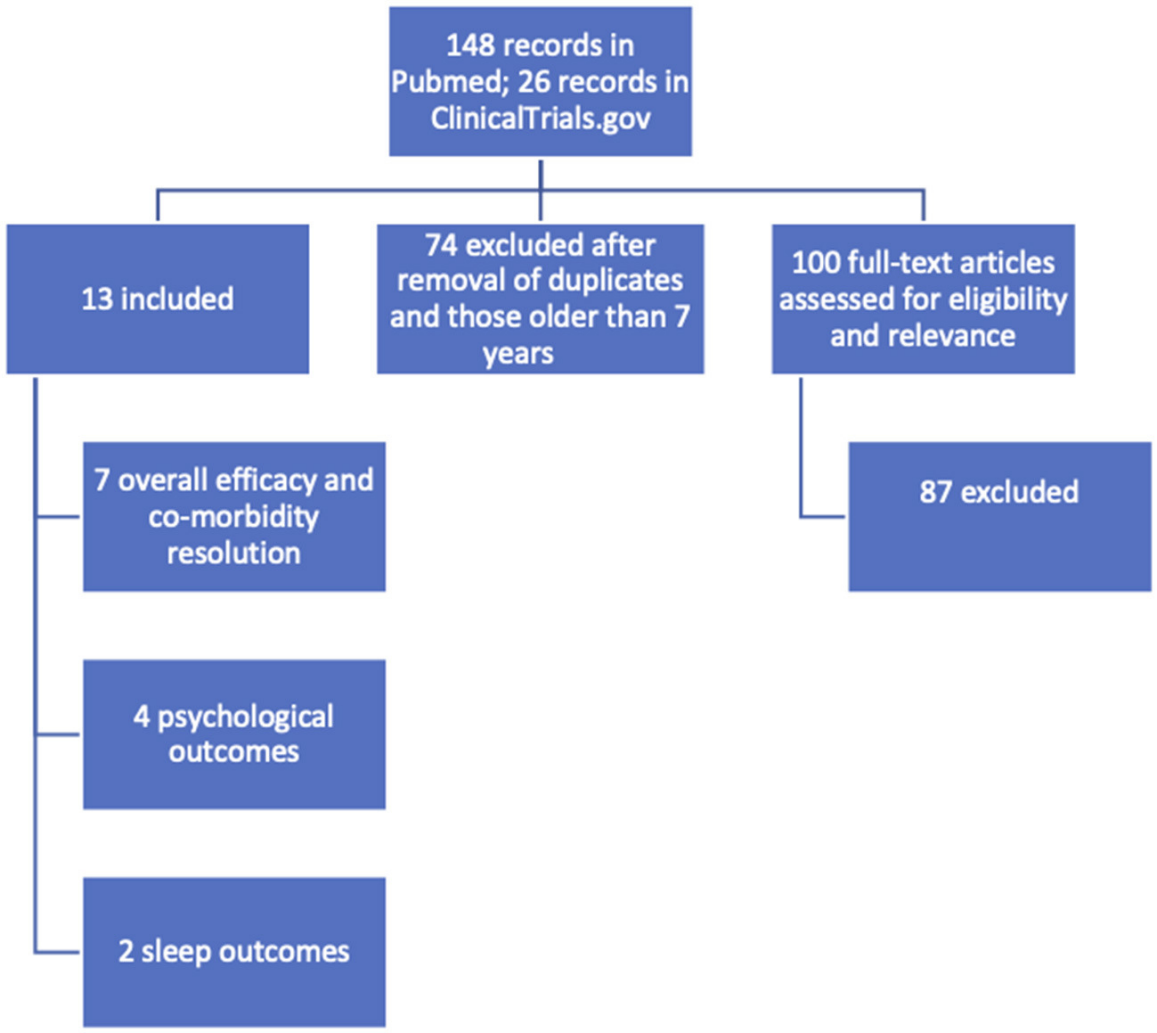
Search and identification scheme for primary studies.

**Figure 2:**
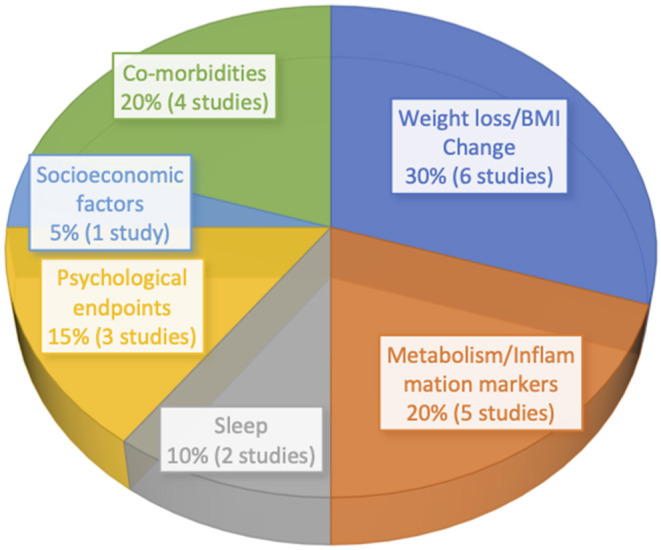
Primary study endpoints by percentage.

For this review, a search was conducted to compile recent data on bariatric surgery outcomes in adolescent patients with regard to weight loss, co-morbidity remission or resolution, sleep, and psychology (Figures 1, 2). Race and socioeconomic status were also examined. Primary studies that examined subjects aged 0–20 and conducted within the past 5 years were considered, and both short- and long-term outcomes were evaluated, using search terms including “bariatric surgery in adolescents” and “obesity in adolescents.” Both single and multi-center studies were considered. Though an attempt was made to include studies that used the same or similar metrics for assessing outcomes, measurement variation between studies made doing so impractical. Thirteen primary studies were evaluated, encompassing a total of 1,287 adolescents (Table 1). Baseline age and BMI ranged from 14 to 19 years and 35 to 91, respectively. The dominant gender was female, and sleeve gastrectomy and Roux-en-Y gastric bypass were the most-performed procedures.

**Table 1:** Study characteristics: primary studies reviewed^a^.

**References**	**Time period**	**Number of subjects (N)**	**Age (years; mean/range)**	**Design**	**Endpoints**	**Limitations**
**Efficacy/co-morbidity resolution**						
Butte et al. (39)	12 months	11 obese; 5 control	16.5 ± 0.8	Prospective cohort	TEE, activity EE, sleep EE, BMR, walking EE	Short-term; small study sample size
Inge et al. (33)	3 years	242	17 ± 1.6	Prospective cohort [Data from Teen-LABS (39, 40)]	Change in body weight, co-morbidities, cardio-metabolic risk factors, and quality of life	Majority female; majority white
Inge et al. (26)	2 years	Surgical: 30 Non-surgical: 63	16.3 ± 1.3 (Surgical); 15.3 ± 1.3 (Non-surgical)	Secondary analysis: Teen-LABS (surgical) and TODAY (non-surgical controls) (39–41)	Change in HbA1c levels, change in BMI (kg/m^2^)	Majority female; small sample size
Kelly et al. (31)	12 months	Cohort 1: 39 Cohort 2: 13	Cohort 1: 16.5 ±1.6 Cohort 2: 16.5 ±1.6	Longitudinal cohort	Change from baseline of IL-6, TNF-alpha, MCP-1, oxLDL, adiponectin, leptin, and resistin	Short duration; majority female; small sample sizes
Klebanoff et a. (4)	3 years	228	17 ± 1.6	State transition model (Markov model; data from Teen-LABS) (39, 40)	QALY, total cost (dollars), incremental cost effectiveness rates	Need for 5-year follow-up; assumption of stable BMI in non-surgical subjects; surgical methods not modeled separately
Ryder et al. (28)	2 years	242	≤ 19	Prospective, multi-center Observational (Data from Teen-LABS) (39, 40)	400 m walk test: Time to complete, resting heart rate, immediate posttest heart rate, and heart rate difference (resting-posttest)	Observational design; lack of non-surgical control group; 400 m walk test not standardized and may not be generalizable
Olbers et al. (42)	5 years	81	16.5 (15.3–17.7)	Prospective, non-randomized, controlled; Multi-site	Weight loss, BMI reduction, co-morbidity resolution	Non-randomized
**Psychology**						
Hunsaker et al. (43)	2 years	Surgical: 139 Obese controls: 83	Surgical: 16.86 Control: 16.11	Controlled, multi-site, sample design (Data from Teen-LABS) (39, 40)	Prevalence, change, predictors, and correlates of psychopathology and associated weight loss	Self-report (questionnaires)
Jarvholm et al. (44)	2 years	88	13–18	Controlled, multi-site, sample design	Symptoms of anxiety, depression, anger, disruptive behavior	Self-report (questionnaires); majority female
Schmitt et al. (45)	2 years	16	17.4 (16.1–18.1)	Prospective	BMI, weight loss, glucose tolerance, triglycerides, hypertension, sleep apnea, cholesterol, fatty liver, quality of life, psychological assessment	Small sample size; male-to-female ratio of 4:12
Zeller et al. (46)	6+ years	14	16 ± 1.3	Observational (2 sequential studies)	BMI (kg/m^2^), weight-related quality of life, mental health, adaptive functioning, substance abuse	Observational design; small sample size; majority female
**Sleep**						
Amin et al. (38)	5 weeks	Surgical: 7 Control: 31	17.8	Prospective cohort	AHI change (events/hr), leptin and orexin levels	Small sample size; short duration; majority male
Zitsman et al. (34, 47)	24+ months	137	17.0 ± 1.2	Prospective cohort	BMI reduction (kg/m^2^), %EWL, Co-morbidity resolution (Type 2 diabetes, OSA, dyslipidemia, PCOS, insulin resistance)	Majority female; lower commitment to follow-up; self-report data for OSA

a *Activity EE, activity energy expenditure; AHI, apnea hypoxia index; BMI, body mass index; BMR, basal metabolic rate; HbA1c, glycated hemoglobin; IL-6, interleukin 6; MC4R, melanocortin 4 receptor; MCP-1, monocyte chemoattractant protein 1; OSA, obstructive sleep apnea; oxLDL, oxidative low density lipoprotein; PCOS, polycystic ovarian syndrome; PYY, peptide YY; QALY, quality-adjusted life years; sleep EE, sleep energy expenditure; TEE, total energy expenditure; TNF-alpha, tumor necrosis factor alpha; walking EE, walking energy expenditure; %EWL, percent excess weight loss; %WL, percent weight loss*.

## Results

Overall, significant decreases in weight and BMI were noted across studies, as were beneficial hormonal alterations (Tables 2–4). In terms of co-morbidities, obstructive sleep apnea (OSA), osteoarthritis, type 2 diabetes mellitus, hypertension, and kidney dysfunction showed significant resolution of up to 95% in some cases; while dyslipidemia, depression, and anxiety showed modest or variable improvement (Tables 2–4). Long-term data beyond 6 years was not available, and data beyond 2 years was limited, highlighting the need for further longitudinal examination in this area, particularly concerning developmental and metabolic changes that occur from adolescence to adulthood.

**Table 2:** Study outcomes and complications: primary studies reviewed^a,b^.

**References**	**Surgical method**	**Primary results**	**Complications**
**Efficacy/Co-morbidities**			
Butte et al. (39)	RYGB	- Significant weight loss - TEE, activity EE, sleep EE, and walking EE significantly declined - Significant decreases in serum insulin, leptin, and T3, gut hormones, and urinary norepinephrine - Significant improvement in BMI (kg/m^2^), body composition, and waist circumference observed only in the surgical intervention group	None noted
Inge et al. (33)	RYGB (161); VSG (67); LAGB (14)	- Significant weight loss - Remission of type 2 diabetes in 95% of participants, remission of abnormal kidney function in 86%, remission of prediabetes in 76%, remission of elevated blood pressure in 74%, remission of dyslipidemia in 66% - Improvement in weight-related quality of life.	- Hypoferritinemia found in 57% of participants at 3 years post-surgery - 13% underwent at least one intraabdominal revision procedure
Inge et al. (26)	RYGB (66%); VSG (28%); LAGB (6%)	- Significant decrease in HbA1c in surgical; increase in non-surgical - Significant decrease in BMI (kg/m^2^) in surgical; increase in non-surgical	- Major complications in 19 patients (8%) - Minor complications in 36 patients (15%)
Kelly et al. (31)	RYGB; VSG	- Leptin and oxLDL decreased significantly; adiponectin increased significantly	None noted
Klebanoff et al. (4)	RYGB (66%); VSG (28%); LAGB (6%)	- QALY gain of 0.199 at 3 years post-surgery. - Cost-effectiveness observed beginning at 5 years	None noted
Ryder et al. (28)	RYGB (161); VSG (67); LAGB (14)	- Significant improvements in walk test time, resting heart rate, and post-test heart rate - Significant improvement in musculoskeletal pain	None noted
Olbers et al. (42)	RYGB	- Significant decrease in BMI (kg/m^2^) - Significant weight reduction (kg)	− 25% underwent additional surgery for complications or rapid weight loss - 72% experienced nutritional deficiencies
**Psychology**			
Hunsaker et al. (43)	RYGB (161); VSG (67); LAGB (14)	- Most subjects retained symptomatic or non-symptomatic status from baseline to 2 years - Remission was more common than development of new symptomology - Preoperative, postoperative, and change in symptoms were unrelated to 2-year percent weight loss.	None noted
Jarvholm et al. (44)	RYGB	- Anxiety, depression, disruptive behavior, and anger decreased significantly at 2 years - Self-esteem, self-concept and mood improved significantly at 2 years	None noted
Schmitt et al. (45)	LAGB	- Significant decrease in BMI (kg/m^2^) - All obesity-related comorbidities improved within the first year post-LAGB - Health-related quality of life showed a trend toward improvement of every physical and psychosocial dimension tested at 1 year, but not statistically significant.	- Delayed wound healing at chamber insertion site. - Eleven patients (69%) experienced one or more complications after LAGB but none severe
Zeller et al. (46)	RYGB	- Both remittance and continued symptomology noted - No new cases of mental health vulnerability emerged	None noted
**Sleep**			
Amin et al. (38)	VSG (6); RYGB (1)	- Significant post-operative decline in AHI (events/hr) at 5 weeks. - Significant decrease in leptin and increase in orexin levels by 3 weeks	None noted
Zitsman et al. (34, 47)	LAGB	- Significant reduction in BMI (kg/m^2^) at 1 year - Significant reduction in AHI (events/hr) at 1 year - Significant percent change in BMI at 5 years - Significant %EWL at 5 years	- 30 patients underwent at least one additional surgery - 27 patients switched to other methods or had bands removed

a *Activity EE, activity energy expenditure; AHI, apnea hypoxia index; BMI, body mass index; BMR, basal metabolic rate; HbA1c, glycated hemoglobin; IL-6, interleukin 6; LAGB, laparoscopic adjustable gastric banding; MC4R, melanocortin 4 receptor; MCP-1, monocyte chemoattractant protein 1; OSA, obstructive sleep apnea; oxLDL, oxidative low density lipoprotein; PCOS, polycystic ovarian syndrome; PYY, peptide YY; QALY, quality-adjusted life years; RYGB, roux-en-Y gastric bypass; sleep EE, sleep energy expenditure; TEE, total energy expenditure; TNF-alpha, tumor necrosis factor alpha; VSG, vertical sleeve gastrectomy; walking EE, walking energy expenditure; %EWL, percent excess weight loss; %WL, percent weight loss*.

b *P < 0.05 for statistically significant results*.

**Table 3:** Objective results^a,b^.

**References**	**Surgical method**	**Primary results**
Butte et al. (39)	RYGB	- **Mean weight loss**: 44 ± 19 kg - **TEE, activity EE, BMR, sleep EE and walking EE**: Significant decreases (*p* = 0.001) through 12 months - **Serum insulin**: Decline from 34.5 ± 24.0 μu/mL to 9.1 ± 5.4 μu/mL (*P* = 0.006) - **Substrate utilization**: Increased significantly for carbohydrates, fats, and proteins - **Adiponectin**: Increase from 6,474 ± 2,540 ng/mL to 10,900 ± 4,820 ng/mL (*P* = 0.002) - **CRP**: Decreased significantly (*P* = 0.01)
Inge et al. (33)	RYGB (161); VSG (67); LAGB (14)	- **Total**: Mean weight decreased by 27% (95% confidence interval [CI], 25 to 29) - **RYGB**: Mean weight decreased by 28% (95% CI, 25 to 30) - **VSG**: Mean weight decreased by 26% (95% CI, 22 to 30) - **T2D**: Remission occurred in 95% (95% CI, 85 to 100) - **Abnormal kidney function**: Remission occurred in 86% (95% CI, 72 to 100) - **Prediabetes**: Remission of prediabetes in 76% (95% CI, 56 to 97) - **Hypertension**: Remission of in 74% (95% CI, 64 to 84) - **Dyslipidemia**: Remission in 66% (95% CI, 57 to 74)
Inge et al. (26)	RYGB (66%); VSG (28%); LAGB (6%)	**Mean HbA1c:**- Teen-LABS–Decreased from 6.8% (95% CI, 6.4–7.3%) to 5.5% (95% CI, 4.7–6.3%) - TODAY–Increased from 6.4% (95% CI, 6.1–6.7%) to 7.8% (95% CI, 7.2–8.3%)
		**BMI:** - Teen-LABS–Decreased by 29% (95% CI, 24–34%) - TODAY: Increased by 3.7% (95% CI, 0.8–6.7%)
Kelly et al. (31)	RYGB; VSG	**Cohort 1 (*****n*** **=** **39)** - Mean BMI at baseline, 51.0 ± 9.6 kg/m2 reduced to 37.4 ± 8.1 kg/m2 (−26.7%) and 34.7 ± 7.7 kg/m2 (−32.0%) at 6- and 12 months, respectively. - IL-6: Baseline 2.3 ± 3.4 pg/mL vs. 6 months: 1.1 ± 1.0 pg/mL, *p* < 0.05 and vs. 12 months: 0.8 ± 0.6 pg/mL, *p* < 0.01 - Leptin: Baseline 178 ± 224 ng/mL vs. 6 months: 44.0 ± 33.1 ng/mL, *p* < 0.001 and vs. 12 months: 41.4 ± 31.9 ng/mL, *p* < 0.001 - Adiponectin: Baseline 5.4 ± 2.4 μg/mL vs. 6 months: 10.2 ± 6.0 μg/mL, *p* < 0.001 and vs. 12 months: 13.5 ± 8.9 μg/mL, *p* < 0.001 - oxLDL was significantly reduced: Baseline: 41.6 ± 11.6 U/L vs. 12 months: 35.5 ± 11.1 U/L, *p* = 0.001 **Cohort 2 (*****n*** **=** **13)** - Mean BMI at baseline, 58.7 ± 6.8 kg/m2, was reduced to 46.8 ± 5.4 (−21.4%) and 36.8 ± 6.5 (−37.4%) at 3- and 12 months, respectively. - IL-6: Baseline 1.7 ± 0.9 pg/mL vs. 3 months: 0.8 ± 1.1 pg/mL, *p* = 0.05 and vs. 12 months: 0.4 ± 0.9 pg/mL, *p* < 0.05 - Leptin: Baseline: 92.9 ± 31.3 ng/mL vs. 3 months: 59.2 ± 52.4 ng/mL, *p* < 0.01 and vs. 12 months: 37.3 ± 33.4 ng/mL, *p* < 0.001 - Adiponectin: Baseline: 6.1 ± 2.9 μg/mL vs. 3 months: 12.7 ± 7.7 μg/mL, *p* < 0.01 and vs. 12 months: 15.4 ± 8.0 μg/mL, *p* < 0.001
Klebanoff et al. (4)	RYGB (66%); VSG (28%); LAGB (6%)	- QALY gain of 0.199 at 3 years post-surgery. - Cost-effectiveness observed beginning at 5 years - Bariatric surgery strategy cost $30,747 more than the no surgery strategy - ICER of bariatric surgery vs. no surgery was $154,684 per QALY; decreased to $114,078 per QALY at 4 years, and $91,032 at 5 years
Ryder et al. (28)	RYGB (161); VSG (67); LAGB (14)	- **Walk test time**: Mean, 376 s; 95% CI, 365–388 to 347 s; 95% CI, 340–358; *P* < 0.01 - **Resting HR**: Mean, 84 beats per minute [bpm]; 95% CI, 82–86 to 74 bpm; 95% CI, 72–76 - **Post-test HR**: Mean, 128 bpm; 95% CI, 125–131 to 113 bpm; 95% CI, 110–116 - **HR difference**: Mean, 40 bpm; 95% CI, 36–42 to 34 bpm; 95% CI, 31–37 - **Post-test HR at 12 months**: Mean, 113 bpm; 95% CI, 110–116 to 108 bpm; 95% CI, 105–111
Olbers et al. (42)	RYGB	- **Weight change**: 36·8 kg (95% CI −40·9 to −32·8) - **BMI**: −13·1 kg/m2 (95% CI −14·5 to −11·8)
Hunsaker et al. (43)	RYGB (161); VSG (67); LAGB (14)	- **Mean weight loss**: 30% - **YSR**: Most subjects retained symptomatic or non-symptomatic status from baseline to 2 years - **YSR**: Remission was more common than development of new symptomology - **YSR**: Preoperative, postoperative, and change in symptoms were unrelated to 2-year percent weight loss (0–24 months; *r* = −0.09, *p* = 0.10)
Jarvholm et al. (44)	RYGB	- Beck Youth Inventory (BYI) scores (Baseline to 2 years)- **Anxiety**:14.2 (12.1–16.4) to 10.5 (8.5–12.7) - **Depression**: 14.1 (12.0–16.6) to 9.9 (7.8–12.4) - **Anger**: 11.3 (9.4–13.5) to 7.8 (5.8–10.1) - **Disruptive behavior**: 4.8 (3.7–6.0) to 3.4 (2.5–4.5) - **Self-esteem and self-concept**: One year post-surgery: 34.6 to 41.3; Two years post-surgery: 40.5
Schmitt et al. (45)	LAGB	- **Median %EWL**: 13.8% ([6.1–58.5], *n* = 16) at 1 month; 40% between 6 months and one year (39.8% [14.2–95.6], *n* = 16); 49.2% ([17.1–98.1], *n* = 10) at 2 years - **BMI decrease**: 40.6 kg·m−2 at day 0 to 36.2 kg·m−2 ([23.5–42.4], *n* = 16) at 1 year; 33.0 kg·m−2 ([23.1–42.7], *n* = 10) at 2 years - **Median fasting insulinemia decrease**: 16.6 μU/mL [7.4–55.1] to 10.1 μU/ml [4.1–15.2] at 12 months (*p* = 0.006) and 7.0 μU/ml [5.3–10.8] at 2 years (*p* = 0.004) - **Median insulinemia at 2 h after OGTT**: Decreased from 80 μU/ml [14.7–220] to 45.5 μU/ml [14–72] after 1 year (*p* = 0.01) - **HOMA-IR decrease**: 3.02 [1.6–10.3] to 1.9 [0.8–3.4] at 12 months and 1.3 [1.1–2.1] at 24 months (*p* = 0.004) - All obesity-related comorbidities improved within the first year - Health-related quality of life showed a trend toward improvement of every physical and psychosocial dimension tested at 1 year, but not statistically significant.
Zeller et al. (46)	RYGB	- Both remittance and continued symptomology noted - No new cases of mental health vulnerability emerged
Amin et al. (38)	VSG (6); RYGB (1)	- **AHI**: decreased by 9.2 events/hour (95% CI: 3.8 to 14.5) - Significant decrease in leptin and increase in orexin levels by 3 weeks
Zitsman et al. (34, 47)	LAGB	- **%EWL**: 35.5 percent (39.4% standard deviation) - **BMI (percent decrease):** 36.6 (37.5% standard deviation) at 5 years. - **AHI**: Significant reduction in AHI (events/hr) at 1 year

a *Activity EE, activity energy expenditure; AHI, apnea hypoxia index; BMI, body mass index; BMR, basal metabolic rate; HbA1c, glycated hemoglobin; IL-6, interleukin 6; LAGB, laparoscopic adjustable gastric banding; MC4R, melanocortin 4 receptor; MCP-1, monocyte chemoattractant protein 1; OSA, obstructive sleep apnea; oxLDL, oxidative low density lipoprotein; PCOS, polycystic ovarian syndrome; PYY, peptide YY; QALY, quality-adjusted life years; RYGB, roux-en-Y gastric bypass; sleep EE, sleep energy expenditure; TEE, total energy expenditure; TNF-alpha, tumor necrosis factor alpha; VSG, vertical sleeve gastrectomy; walking EE, walking energy expenditure; %EWL, percent excess weight loss; %WL, percent weight loss*.

b *P < 0.05 for statistically significant results*.

**Table 4:** Primary surgical outcomes^a,b^.

	**Mean (95% CI) change**	**95% CI**	**No. of studies reporting**	**Notes**
BMI	29% decrease	24–37.5%	4	Duration 1–5 years
Weight loss	27% decrease	25–29%	4	Duration 1–5 years
%EWL (1-2 years post)	90.5%	85–96%	2	Percent excess weight loss (%EWL) 1 to 2 years post-surgical procedure
%EWL (4-5 years post)	41.5%	36–47%	1	4-5 years post-surgical procedure
Hemoglobin A1c	19% decrease from baseline (6.8 to 5.5%)	Baseline: 6.4–7.3% Decrease: 4.7–6.3%	1	In diabetic patients
Co-morbidities (Type 2 diabetes, dyslipidemia, kidney disease, OSA, osteoarthritis, HTN, NAFLD)	80.5% reduction	66–95%	4	From baseline
Energy Expenditure (TEE, activity EE, sleep EE, BMR, walking EE)	*Significant decrease (*p* = 0.001)		1	From baseline to 1 year
QALY	Gain of 0.199	2.057 (no surgery) to 2.256 (surgery)	1	At 3 years
Metabolic/Inflammatory markers				
Serum insulin	73.6% decrease	58.2–89.6%	2	At 1 year
Adiponectin	41.3% increase	36.3–72.5%	2	At 1 year
Leptin	76.7% decrease	59.4–94.7%	1	At 1 year
oxLDL	14.7% decrease	−12–41.5%	1	At 1 year
IL-6	65.2% decrease	−27–81.8%	1	At 1 year
Cardiology				
Walking time	7.7% decrease	4.8–9.6%	1	At 1 year
Resting heart rate	11.9% decrease	9.5–14.3%	1	At 1 year
Post-test heart rate	4.4% decrease	1.8–7.0%	1	At 1 year
Heart rate difference	15.0% decrease	7.5–22.5%	1	At 1year
Psychological	**Baseline to 2 years using IWQOL-Kids, BDI, and perceived confidence**			
Anxiety	26%	22.6–29.7%	1	Baseline to 2 years
Depression	29.8%	25.3–35%	1	Baseline to 2 years
Disruptive behavior	29.2%	25–32.4%	1	Baseline to 2 years
Anger	30.9%	25.6–38.3%	1	Baseline to 2 years
Mood	*Sig increase		1	Stabilization at 2 years
Self-esteem	*Sig increase		1	Stabilization at 2 years
Self-concept	*Sig increase		1	Stabilization at 2 years

a *BMI, body mass index; HbA1c, glycated hemoglobin; oxLDL, oxidative low density lipoprotein; %EWL, percent excess weight loss; QALY, quality-adjusted life years; sleep EE, sleep energy expenditure; TEE, total energy expenditure; walking EE, walking energy expenditure*.

b *P < 0.05 for statistically significant results*.

### Weight Loss

The mean (95% CI) decrease in BMI across studies in which this endpoint was assessed was 29% (24–37.5%). Mean weight loss, where measured, was 27% (25–29%). Mean percent excess weight loss (%EWL), when reported, was 90.5% (85–96%) for 1 year post-surgical procedure, and 41.5% (36–47%) at 3 to 5 years post-surgical procedure.

### Co-morbidity Resolution/Outcome

The mean (95% CI) decrease in hemoglobin A1c concentration in diabetic patients was from a baseline of 6.8% (6.4–7.3%) to 5.5% (4.7–6.3%). Additionally, including type 2 diabetes, among the studies that measured overall co-morbidity outcomes, the mean percent (95% CI) resolution of such conditions from baseline was 80.5% (66–95%). Such co-morbidities included dyslipidemia, kidney disease, OSA, osteoarthritis, hypertension, and non-alcoholic fatty liver disease.

### Psychological Endpoints

Most patients retained symptomatic or non-symptomatic status from baseline to 2 years, and remission was more prevalent than the development of new symptomology across studies. Anxiety, depression, disruptive behavior, and anger all decreased significantly at 2 years, contrasted by increases in mood, self-esteem, and self-concept at the same time point, reflected in IWQOL-Kids, BDI, and perceived confidence scores.

## Bariatric Surgery: Overview of Procedures and Risks

Three primary methods of bariatric surgery are commonly performed: Roux-en-Y gastric bypass, sleeve gastrectomy, and the adjustable gastric band. Among these, gastric bypass is generally considered the “gold standard,” with the bulk of evidence in the long- and short-term landing in favor of the procedure for its safety and efficacy (27, 48). In the gastric bypass procedure, a 30-milliliter stomach pouch is created by dividing the stomach in half, to which surgeons connect a portion of the small intestine, which has also been divided (48). With a smaller stomach, less food is consumed; and with a shortened small intestine, fewer nutrients are absorbed (48). Additionally, the altered digestive route significantly impacts gut hormones such as ghrelin, leptin, and glucagon-like peptide 1 (GLP-1), resulting in suppression of hunger and stimulation of satiety (48). Those who are not candidates for gastric bypass due to BMI restrictions or severity of co-morbidities, usually undergo a sleeve gastrectomy, which removes 80 percent of the stomach (48), and has been shown to be more effective than the adjustable gastric band (27). In adjustable gastric banding, a reversible procedure, surgeons install an inflatable band around the upper stomach. This leaves only a small area of stomach above the band, thereby reducing the quantity of food the stomach can accommodate (48). Gastric banding, however, has decreased in popularity due to the more dramatic results of the bypass and sleeve methods.

A young person is eligible for bariatric surgery if he/she has a BMI >40, or a BMI >35 with associated clinical illness(es), such as type 2 diabetes, hypertension, chronic liver disease, obstructive sleep apnea (OSA), or cardiac deficits (22, 23, 49). Additionally, most surgical centers require psychological evaluation lasting 1 month or longer to determine the patient's ability to adhere to dietary guidelines and follow-up after surgery (49), and the patient's insurance must pre-authorize the procedure, creating a barrier for some patients. The most common procedures in these patients are the sleeve gastrectomy and gastric bypass, and patients must have a full team in place prior to surgery, including the surgical staff, nutritional support, psychological support, and an exercise specialist (49).

Risks associated with bariatric surgery in adolescents are magnified by the extent of related co-morbidities, and multiple studies support early intervention to minimize risk of complications (22, 23, 33, 49). Following surgery, patients may face absorption defects, particularly involving micronutrients such as B-vitamins, vitamin D, and calcium, highlighting the necessity for proper nutritional support from family members and specialists (49). Patients should consider ongoing psychological care following surgery, as there is not yet enough long-term data to rule out adverse mental health ramifications (49), or to rule in significant positive change.

## Discussion

### Physiological Effects of Bariatric Surgery in Adolescents: Experimental Findings

The dramatic efficacy of bariatric surgery on both weight loss and co-morbidity resolution that is well-documented in adult patients is echoed in available research for children and adolescents. Studies show weight loss of up to 96 percent of excess body weight in the short-term (up to 1 year) and 39.4 percent sustained in the longer-term (up to 6 years), complete resolution or substantial improvement in type 2 diabetes, decreased appetite and increased satiety, and significant resolution of musculoskeletal pain (26, 28–33).

One large-scale, longitudinal assessment currently being conducted is Teen-LABS (40, 50), a consortium comprised of six clinical sites and a data-coordinating center, gathering information from participating clinical sites to develop a database of surgical outcomes in adolescents. Primary measured surgical outcomes include percent change in BMI, and remission from baseline type 2 diabetes and hypertension, with secondary measured outcomes being incidence of iron and B12 deficiency, and occurrence of abdominal reoperations.

Many of the studies reviewed herein utilize data currently available from this project (4, 26, 28, 33, 46). In one such study, Inge et al. (33) reported a decrease in mean weight of 27 percent, a decrease in kidney dysfunction in 86 percent of patients who had such dysfunction at baseline, remission of hypertension in 72 percent, and resolution of dyslipidemia in 66 percent. Weight-related quality of life also showed significant improvement. The researchers did find an increase in iron deficiency in over half of the participants, and 13 percent underwent revision surgeries. This study looked at outcomes after 3 years of follow-up; consequently, it is one of the longer-term sources of data available.

Inge et al. also performed a secondary study looking exclusively at adolescents with type 2 diabetes, comparing data from Teen-LABS to that from TODAY, a study in which participants were randomized to receive metformin therapy only, metformin therapy in combination with rosiglitazone, or an intensive lifestyle intervention (26, 51). A cohort of 30 subjects in the Teen-LABS study showed a decrease in mean hemoglobin A1c concentration from 6.8% at baseline to 5.5% over the course of 2 years, while that in the 63 subjects in the TODAY study increased from 6.4 to 7.8% (26). Further, mean BMI in the Teen-LABS group decreased by 29%, while mean BMI in the TODAY group increased by 3.7% (26). These results offer compelling support for bariatric surgery in these cohorts; however, for accurate comparison, it is necessary to examine prospective studies to eliminate possible confounders and biases.

One such prospective cohort, and another source of long-term data is the ongoing study by Zitsman et al. which recently posted 5-year follow-up data for all 137 study participants regarding BMI and %EWL endpoints (47). Researchers found a mean post-laparoscopic banding decrease in %EWL of 35.5 percent (39.4% standard deviation), and a percent BMI decrease of 36.6 (37.5% standard deviation) at 5 years. Similar results are reported by Olbers et al. in a Swedish nationwide study that also followed participants over 5 years; however, in this cohort, the surgical method evaluated was Roux-en-Y gastric bypass (42), rather than gastric banding. Participants in this study had a mean (95% CI) weight reduction of 36.8 kg (32.8–40.9 kg), and a mean decrease in BMI of 13.1 kg/m^2^ (14.5–11.8 kg/ m^2^). The study also reported significant co-morbidity resolution and a decrease in cardiovascular risk factors over the 5 years; however, researchers noted weight loss of <10 percent in nine participants (11%). These longitudinal studies reflect an even greater degree of efficacy than previously seen in Teen-LABS (40, 50), and again highlight the need for further long-term data.

In a study of 12 months' duration, Kelly et al. (31) reported a significant decrease in leptin levels post-surgical procedure, with a concurrent increase in adiponectin levels but no significant change in resistin levels. Further, researchers found that interleukin-6 (IL-6) levels decreased significantly, as did oxidative LDL cholesterol levels, leading to improved lipid profiles. This study population was small, encompassing 52 subjects in two separate cohorts, one of which included participants from Teen-LABS, but the duration suggests that longer follow-up could affect measured hormone outcomes.

In their study examining energy expenditure post-bariatric surgery, Butte et al. (39) noted not only significant decreases in total, activity, resting, and walking energy expenditure, measured by 24-h room calorimetry, but also a parallel and significant decline in serum insulin, and decreases in fasting serum TSH and total serum triiodothyronine (T3), indicating alterations in thyroid function post-surgery. Leptin also decreased significantly, as did urinary norepinephrine associated with the alterations in energy expenditure and basal metabolic rate (BMR), and substrate utilization increased significantly for carbohydrates, fats, and proteins. Additionally, adiponectin increased, and levels of inflammatory marker CRP decreased significantly (*P* = 0.01). No significant changes were seen in resistin levels, echoing results produced by Kelly et al. Again, this study had a small population of only 11 subjects and five controls; however, the findings at 1 year closely mirror those seen previously, strengthening the evidence for improved energy and metabolic profiles post-bariatric surgery in these young patients, and suggesting the need for further study.

### Psychology in the Obese Adolescent: Experimental and Observational Findings

Recent evidence suggests that bariatric surgery has a significant and positive impact on the psychological co-morbidities of obese teenagers. A study by Schmitt et al. examining the outcomes of laparoscopic adjustable gastric banding, in addition to noting positive physiological outcomes (Tables 1–3), also gathered data on the perceived reasons for the onset of obesity in the adolescent patients (45). According to the responses, more than half of the participants attributed the start of their weight gain to a single, deleterious life event (45), such as divorce or a parental suicide, and the same proportion presented with a psychiatric history at baseline. In addition, study participants noted teasing (*n* = 11), decreases in self-esteem (*n* = 7), body dissatisfaction (*n* = 14), anxiety (*n* = 5), and attempted suicide (*n* = 1) as triggers of current psychological distress. This study found an improvement in depressive symptoms and social isolation at 1 year post-surgical intervention, and results from Psychosocial PedsQL evaluations noted statistically significant improvement at both 1 and 2 year endpoints in emotional functioning, social functioning, and school functioning (45). However, the study was performed in a specialty obesity center with a cohort of only 16 subjects, so it is not necessarily generalizable to the population as a whole.

Jarvholm et al. examined a cohort of Swedish adolescents, aged 13–18, in an attempt to determine if the positive psychological outcomes observed in research available for adult patients were mirrored in these younger patients at 2-years post-surgery (44). According to Jarvholm et al. (44), anxiety, depression, anger, and disruptive behavior all decreased significantly at 2 years post-bariatric surgery in a cohort of 88 adolescents, using Beck Youth Inventory (BYI) scores. Additionally, self-esteem and self-concept improved significantly at 1 year post-surgery, with stabilization occurring by the 2-year mark.

Hunsaker et al. (43) found less dramatic improvement over 2 years, with most of the subjects maintaining their symptomatic psychological status; however, remission or improvement was more common than the occurrence of new symptomology. These findings were mirrored by Zeller et al. (46); however, this study utilized Youth Self-Report (YSR) and Adult Self-Report (ASR) examinations, which have the potential to introduce bias. The study had a follow-up period that lasted as long as 6 years, however, making it one of the longest-term studies currently available for analysis. It is important to note the difficulty of evaluating psychological improvement, as studies measure according to clinical diagnosis, which does not always apply uniformly across individuals. Further, self-reported methods are often employed, resulting in potential bias. More study, particularly long-term and objective, is needed to clearly assess the mental health changes, though initial results in both adult and adolescent studies lend strong evidence to the efficacy of bariatric surgery on psychological endpoints.

### Sleep Quality and Disorders in the Obese Adolescent: Experimental Findings

Sleep disorders such as obstructive sleep apnea (OSA), circadian rhythm disorders, and poor sleep quality show a high prevalence in obese adolescents (17). He et al. found that habitual sleep variability is significantly associated with abdominal and visceral obesity (17), and even more compelling, that 20 percent of the association with visceral adiposity can be attributed to carbohydrate intake, an effect more clearly delineated in a recent review by St-Onge et al. (18). Additionally, due to a delay in melatonin release during the adolescent years, teenagers are more prone to sleep restriction resulting from the delayed sleep onset, combined with the early start of the typical school day (17). Such short sleep duration can lead to weight gain and eventual obesity, due to an increase in neuronal reward responses to unhealthy foods, potential alterations in hormonal activity (leptin, ghrelin, GLP-1, orexins), and the increased time awake that leads to higher caloric intake (18–20). Similarly, in a randomized, crossover, sleep restriction-extension paradigm, Beebe DW et al. reported that adolescents with chronic sleep restriction tend to consume high glycemic index foods, and that such sleep restriction could cause lasting changes in dietary habits that increase the risk of obesity into adulthood (52).

Many obese adolescents also experience varying degrees of OSA (37), and according to findings by Talib et al. OSA is closely related to BMI Z-score (3.7 vs. 3.4; *P* = 0.003) (53).

Mounting evidence suggests that bariatric surgery is instrumental in reversing or minimizing the impact on sleep caused by obesity (36, 37), and can even be beneficial in stabilizing hormonal factors that interfere with proper sleep structure (38). According to findings by Amin et al. (38), apnea hypoxia index (AHI) decreased post-surgery from 15.8 events per hour at baseline to 9.1 events per hour at 5 weeks post-surgery. In the same cohort, leptin levels decreased significantly and orexin levels increased significantly, demonstrating an early alteration in hormone activity following surgical intervention. Ashrafian et al. (37) found similar improvements in OSA in their recent meta-analysis, noting significantly stronger results in those who received bariatric surgery vs. traditional weight loss interventions. In one of the few studies examined that focused on gastric banding, Zitsman et al. (34) utilized polysomnography to diagnose 35.8% of study subjects (49 patients) with OSA, and the researchers noted a resolution of OSA in 17 of those patients post-surgery. It is important to note, however, that complication rates in this study were more prevalent than in studies investigating sleeve and bypass techniques, and improvement in OSA was measured by self-report rather than formal re-testing. Few studies are available that examine the changes in OSA following bariatric surgery in adolescents specifically—and even fewer look at the long-term efficacy; consequently, more data is needed prior to definitive conclusions regarding the positive outcomes noted here.

### Race, Ethnicity, and Socioeconomic Status: Experimental and Epidemiological Findings

As with the vast majority of medical procedures, socioeconomic status (SES) plays a role in patient eligibility due to quality of insurance, cost of support structures and specialists, proximity to high-quality facilities, and availability of transportation/lodging when necessary. Because obesity has been strongly linked to those of lower SES and has a disproportionate impact on Black and Hispanic adolescents when compared with non-Hispanic whites (11, 41, 54–56), considering not only SES, but also race, ethnicity, and geographic location is important when examining the accessibility of bariatric surgery for some of the most at-risk populations. In reviewing the current literature, very few studies specifically focus on these areas with regard to bariatric surgery outcomes in adolescents.

Gullick et al. (56) examined both race and SES in relation to bariatric surgery outcomes, finding similar improvements in co-morbidities, but lower weight loss in African American subjects than European Americans at 1, 2, and 5 year endpoints. The study, however, enrolled 74.4 percent European Americans, and only 25.6 percent African Americans, and it also studied adult, rather than adolescent, subjects. Based on the evidence, it appears that SES may be a bigger predictor of outcomes than ethnicity; however, not enough data exist to make a definitive conclusion (55, 56). What can be assessed is that those of lower SES or living in rural areas further from reputable medical centers, have more post-operative complications and lower success rates than those with better access to resources (11).

Perez et al. found that age-adjusted severe obesity rates were higher in minority adolescent groups: black adolescents have a rate of 8.6% and Hispanic adolescents a rate of 8.4%, while white adolescents have less than half that rate at 4.1% (*P* = 0.03) (57). The researchers also found that white adolescents utilize bariatric surgery at a much higher rate than black or Hispanic patients, and that there is a paradoxical relationship with regard to insurance type (57): minority patients are even less likely to undergo weight loss surgical procedures if they carry Medicaid rather than private insurance; however, white patients with Medicaid are much more likely to undergo such procedures than those insured by private carriers. Though the reasons for such dichotomy are as of yet unclear, what Perez et al. pointed out was that the solution is far more complex than a simple expansion of government-sponsored health insurance. As with many complicated issues, there are several components at play here, including cultural context/norms, health education, and physical access to care. Each must be examined specifically within the context of the adolescent population to determine future public health measures that could mitigate such disparity and ensure a more equitable distribution of care.

## Conclusion

For decades, bariatric surgery has been considered a highly effective treatment method for adults with morbid obesity and related co-morbidities; however, efficacy and safety in children and adolescents, particularly with regard to metabolic profiles, psychology and sleep disorders, has not been adequately evaluated, especially for long-term outcomes. The data presented in this review lends further support to the continued use of bariatric surgery in adolescents, with an emphasis on the need for further long-term data and more extensive data on metabolic changes in this population post-surgery. In compiling this review, it is important to note the limitation in making adequate comparisons, as metrics used to determine endpoints of obesity, such as BMI, percent excess weight loss (%EWL), percent weight loss (%WL), and total weight loss, are not uniformly measured across studies. Additionally, this review examined a wide range of factors regarding bariatric surgery and adolescent obesity, though a lengthy review could easily be written for each individual aspect. As the obesity epidemic shows no signs of decline, findings in this area will become increasingly vital to health care professionals and policy makers alike.

Note: The BASIC trial, the first randomized, controlled trial looking at bariatric surgery as a treatment for adolescents for whom lifestyle interventions have failed, is in its early stages, and could provide useful long-term data in the future, including insights into psychosocial outcomes (58). Additionally, several clinical trials and prospective cohort studies are currently pending completion, recruiting, or generating results. Findings from these projects could yield the much-needed information regarding the long-term safety and efficacy of bariatric surgery in younger patients that this paper has found to be currently lacking.

## Author Contributions

CR designed and conducted research, analyzed data, wrote the paper, primary responsibility for final content, read, and approved the final manuscript.

## Conflict of Interest

The author declares that the research was conducted in the absence of any commercial or financial relationships that could be construed as a potential conflict of interest.

## Abbreviations

ADHD: Attention deficit hyperactivity disorder
AHI: Apnea hypoxia index
BMI: Body mass index
BMR: Basal metabolic rate
GLP-1: Glucagon-like peptide 1
IL-6: Interleukin-6
LDL: Low-density lipoprotein
MC4R: Melanocortin 4 receptor
OSA: Obstructive sleep apnea
oxLDL: Oxidative low-density lipoprotein
PYY: Peptide YY
QALYs: Quality adjusted life years
SES: Socioeconomic status
T3: Triiodothyronine
WHO: World Health Organization
%EWL: Percent excess weight loss
%WL: Percent weight loss.
